# Pulmonary Function and Nocturnal Hypoxemia Patterns in Patients with Obstructive Sleep Apnea

**DOI:** 10.3390/jcm14103589

**Published:** 2025-05-21

**Authors:** Claudia Lucia Toma, Filip Radu, Dragos-Cosmin Zaharia, Ionela Belaconi, Stefan Dumitrache-Rujinski

**Affiliations:** 1 1st Pulmonology Section, 4th Department—Cardio-Thoracic Pathology, “Carol Davila” University of Medicine and Pharmacy, 020021 Bucharest, Romaniastefan.dumitrache@umfcd.ro (S.D.-R.); 2 4th Pulmonology Department, “Marius Nasta” Institute of Pneumology, 050159 Bucharest, Romania

**Keywords:** obstructive sleep apnea, COPD, pulmonary function, pulse oximetry waveform patterns

## Abstract

**Background/Objective:** Obesity is a documented risk factor for impaired pulmonary function and abnormal oxyhaemoglobin levels during sleep. This functional impairment becomes more significant when there are additional respiratory pathologies, such as obstructive sleep apnea (OSA) and/or chronic obstructive pulmonary disease (COPD). Overnight pulse oximetry may offer an effective evaluation of nocturnal oxyhaemoglobin levels/waveform patterns. We evaluated the correlation between obesity, overnight pulse oximetry (parameters, waveform patterns) and pulmonary function in patients diagnosed with moderate–severe OSA and normal oxyhaemoglobin saturation levels during waking hours. We also compared the overnight oxyhaemoglobin saturation levels between patients with OSA alone and those with associated COPD. **Methods:** This was a retrospective, transversal, non-interventional study on consecutive patients with moderate–severe OSA diagnosed using overnight cardiorespiratory polygraphy over a period of 18 months. After analyzing the study population’s characteristics, the patients were divided into two subgroups: one consisting of patients with OSA alone (Group A), and the second with coexisting OSA and COPD (Group B). **Results:** Seventy-six patients were included in the study, and 18% were diagnosed with COPD. A higher body mass index (BMI) correlated with a higher number of ≥3% SpO_2_ drops/h (ODI3) and percentage of time with oxyhaemoglobin saturation < 90% (t90) and a lower average nocturnal oxyhaemoglobin saturation (avgSpO_2_). ODI3 correlated negatively with avgSpO_2_ and positively with t90. After eliminating BMI as a confounding factor, lower values of forced expiratory volume in the first second (FEV1) were associated with lower avgSpO_2_ and higher t90. FEV1 did not corelate with ODI3. After dividing the study population into the two subgroups, patients from Group B had a tendency towards lower average nocturnal SpO_2_ levels compared to Group A. **Conclusions:** Different phenotypes/patterns of nocturnal hypoxemia can be identified using quantitative and qualitative analyses of overnight pulse oximetry: repetitive, consecutive obstructive respiratory events with a characteristic intermittent (saw-tooth) hypoxemia pattern and alveolar hypoventilation, resulting in a continuous (plateau) hypoxemia pattern. According to our findings, nocturnal hypoxemia is more important at lower FEV1 values (correlating with lower avgSpO_2_/higher t90, but not with ODI3). The presence of a continuous hypoxemia pattern in patients with OSA may suggest that pulmonary function tests should be performed in order to differentiate patients with alveolar hypoventilation secondary to obesity (restrictive syndrome) from those with associated COPD (obstructive syndrome). This can have an impact on the management of the case and the therapeutic approach (positive pressure therapy with/without supplemental oxygen).

## 1. Introduction

Obesity is a well documented risk factor for impaired pulmonary function [1] and abnormal oxyhaemoglobin waveform patterns during sleep [2]. Upper airway collapsibility due to adipose tissue accumulation [3] and the reduced respiratory drive observed in obese patients represent important links in the causal chain of obstructive sleep apnea (OSA) [4]. At the same time, the android-abdominal fat distribution reduces the compliance of the thoracic chest wall, which contributes to the development of alveolar hypoventilation [5]. Some patients may also develop obstructive spirometry patterns due to adipose tissue accumulation in the bronchial wall [6], systemic inflammation secondary to sleep apnea [7] and obesity itself [8].

The functional impairment becomes more significant when there exists an additional pathology, such as chronic obstructive pulmonary disease (COPD). In these patients, emphysema with the destruction of lung parenchyma and alveolo-capillary membrane [9], ventilation–perfusion mismatch due to mucus plugging, bronchospasm and bronchial remodeling [10], hypoxic pulmonary hypertension [11], and systemic inflammation [12] further affect oxyhaemoglobin levels during sleep, even in the presence of normal oxygen saturation levels during waking hours [13].

Moreover, for these patients, heart failure with preserved ejection fraction (HFpEF) secondary to multiple pathophysiological mechanisms, such as hypertensive, dysmetabolic/sleep apnea, and ischemic cardiomyopathy, induces altered hemodynamics in lung circulation [14,15].

Overnight pulse oximetry offers an effective and accessible (inexpensive, non-invasive, repeatable, point of care) evaluation of the nocturnal oxyhaemoglobin saturation in patients with a variety of cardio-pulmonary pathologies [16]. It is most commonly used to diagnose and assess the severity of oxyhaemoglobin desaturation and waveform patterns in patients with OSA, to detect the presence of alveolar hypoventilation, and to quantify the level of hypoxemia in patients with COPD [13].

In addition to a wide range of oximetric (quantitative) parameters, such as average oxyhaemoglobin saturation, time spent with oxyhaemoglobin saturation below 90%, and oxyhaemoglobin desaturation index, pulse oximetry reports also allow for a subjective/qualitative evaluation of nocturnal oxyhaemoglobin saturation patterns/aspects using pulse oximetry waveform tracings [17].

This combined evaluation (quantitative and qualitative assessment of overnight pulse oximetry) is not only useful in characterizing the different phenotypes of nocturnal respiratory failure but can also be a guiding factor in selecting the most appropriate treatment and monitoring the efficiency of positive airway pressure [18].

The main objective of this study was to evaluate the correlation between overnight oximetric parameters and the results of pulmonary function tests in patients diagnosed with moderate and severe obstructive sleep apnea, with normal oxyhaemoglobin saturation levels during waking hours.

We aimed to investigate the relationships between the oxyhaemoglobin desaturation index, obesity, and lung function, and their impact on nocturnal oxyehaemoglobin saturation levels, using overnight pulse oximetry.

This study also compared the overnight oxyhaemoglobin saturation levels between patients with obstructive sleep apnea alone and those with obstructive sleep apnea and associated COPD (overlap syndrome). The purpose was to evaluate lung parenchymal impairment as a possible contributing factor for nocturnal hypoxemia (in patients with associated COPD), in addition to existing intermittent desaturations and hypoventilation due to obstructive sleep apnea and obesity.

Two mechanisms of nocturnal hypoxemia were proposed: intermittent hypoxemia (characteristic of obstructive sleep apnea), and continuous hypoxemia, secondary to nocturnal alveolar hypoventilation due to obesity or COPD.

Concurrent with the quantitative analysis, we also provided the visual/qualitative patterns of the pulse oximetry waveforms (identifying the two above proposed hypoxemia mechanisms): saw-tooth and continuous/plateau patterns, respectively.

Using a combined analysis (quantitative assessment of pulse oximetry parameters and subjective/qualitative analysis of waveform patterns), we aim to characterize the underlying mechanisms of nocturnal hypoxemia present in patients diagnosed with moderate and severe obstructive sleep apnea, presenting with normal oxyhaemoglobin saturation levels during waking hours.

This article is a revised and expanded version of a paper entitled Relația dintre hipoxemia nocturnă, funcția pulmonară și indexul de masă corporală la pacientul cu apnee în somn de tip obstructiv, which was presented at The 28th National Congress of the Romanian Society of Pneumology, Romania, 13–16 November 2024 [19].

## 2. Materials and Methods

We conducted a retrospective, transversal, and non-interventional study. We analyzed data from the medical files of consecutive patients diagnosed with obstructive sleep apnea (OSA) over a period of 18 months (January 2023–June 2024) referred to the 4th Pulmonology Department of the “Marius Nasta” Institute of Pneumology in Bucharest, Romania.

The study was approved by the Ethics Committee of the “Marius Nasta” Institute of Pneumology (4655/11 March 2025).

The inclusion criteria were ≥18 years of age; consent concerning the use of medical data for academic purposes, with the condition of anonymity; positive diagnosis of moderate and severe obstructive sleep apnea (apnea–hypopnea index ≥ 15 events/h), using home overnight cardiorespiratory polygraphy; no daytime hypoxemia; no comorbid respiratory pathologies other than COPD; and complete data regarding the studied parameters. Patients not meeting all of the inclusion criteria were excluded from the study.

For home overnight cardiorespiratory polygraphy, we used the Porti SleepDoc^®^ (Löwenstein Medical, Bad Ems, Germany) 6-channel device (nasal airflow pressure cannula, digital pulse oximeter, respiratory effort belt, body position). All cardiorespiratory polygraphy recordings were manually validated using the AASM Manual for the Scoring of Sleep and Associated Events as a guideline.

Spirometry measurements were performed at the department for pulmonary function tests, using a COSMED^®^ (Albano Laziale, Rome, Italy) spirometer.

The following data were collected for the study: age and body mass index (BMI); smoking history and positive diagnosis of chronic obstructive pulmonary disease (COPD) according to GOLD criteria; spirometry measurement of forced expiratory volume in the first second (FEV1), expressed as a percentage of the predicted value; quantitative results from continuous overnight pulse oximetry (part of the home overnight cardiorespiratory polygraphy investigation), represented by average nocturnal oxyhaemoglobin saturation (avgSpO_2_), percentage of continuous pulse oximetry evaluation time with oxyhaemoglobin saturation below 90% (t90), number of drops with ≥3% SpO_2_ per hour of recording time (ODI3); and overnight pulse oximetry waveform.

Microsoft Excel 2010^®^ and IMB SPSS Software 26^®^ were used for data collection and statistical analysis. Normality tests were applied (Kolmogorov–Smirnov, Shapiro–Wilk) and the data were expressed as mean, standard deviation, median, and interquartile range.

To identify the physiological correlations between anthropometric data, nocturnal oxyhaemoglobin saturation, and pulmonary function, the Spearman correlation was applied to the following variables: BMI, ODI3, avgSpO_2_, t90, and FEV1.

The studied population was then divided into two subgroups: patients with OSA alone (Group A, n = 62) and patients with OSA-COPD overlap (Group B, n = 14).

To test whether there was a meaningful difference in pulmonary function between the two subgroups, Student’s *t*-test was applied to the FEV1 variable.

The Mann–Whitney *U* test was applied to avgSpO_2_ and t90 to test whether there was a significant difference in the nocturnal oxyhaemoglobin saturation between the two subgroups. The test was also used to evaluate whether there was any difference between the two subgroups concerning age, BMI, and OSA severity (quantified by ODI3) that could influence nocturnal oxyhaemoglobin saturation levels.

A *p*-value lower than 0.05 was considered statistically significant. The resulting data were represented using tables and scatter plot graphics.

## 3. Results

After applying the eligibility criteria, 76 patients were included in the study (male–female, M:F 50:26). Out of them, 45 (59%) patients had a history of smoking, and 14 (18%) patients were diagnosed with COPD. The patients were divided into two subgroups: Group A, n = 62, M:F = 40:22, consisting of patients with OSA alone, and Group B, n = 14, M:F = 10:4, consisting of patients with coexisting OSA and COPD (overlap syndrome). The patient characteristics of the studied population and the comparison between the two subgroups are represented in Table 1 and Table 2.

Concerning BMI, there was a significant positive relationship between BMI and ODI3 (*r* = 0.52, *p* < 0.001; Spearman’s correlation), a significant negative relationship between BMI and avgSpO_2_ (*r* = −0.36, *p* = 0.001), and a significant positive relationship between BMI and t90 (*r* = 0.44, *p* < 0.001).

The severity of OSA, as quantified by ODI3, correlated with nocturnal oxyhaemoglobin saturations as follows: significant negative relationship between ODI3 and avgSpO_2_ (*r* = −0.59, *p* < 0.001) and significant positive relationship between ODI3 and t90 (*r* = 0.66, *p* < 0.001).

**Figure 1:**
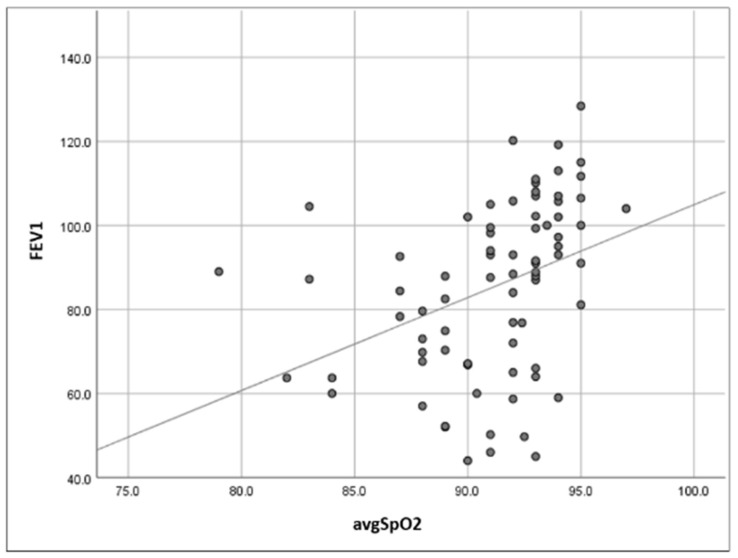
Scatter plot representation of relationship between forced expiratory volume in the first second (FEV1) and average nocturnal oxyhaemoglobin saturation (avgSpO_2_).

**Figure 2:**
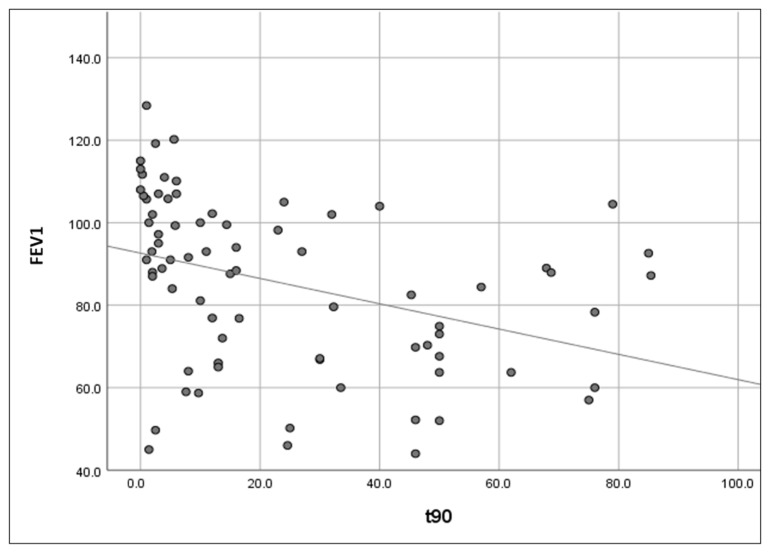
Scatter plot representation of the relationship between forced expiratory volume in the first second (FEV1) and percentage of continuous pulse oximetry evaluation time with oxyhaemoglobin saturation below 90% (t90).

Pulmonary function, as expressed by FEV1, correlated with BMI and nocturnal oxyhaemoglobin saturation as follows: significant negative relationship between FEV1 and BMI (r = −0.27, *p* = 0.015), significant negative relationship between FEV1 and ODI3 (r = −0.31, *p* = 0.006), significant positive relationship between FEV1 and avgSpO_2_ (r = 0.50, *p* < 0.001; Figure 1), and significant negative relationship between FEV1 and t90 (r = −0.50, *p* < 0.001; Figure 2).

After eliminating the confounding factor represented by BMI, there was a significant positive correlation between FEV1 and avgSpO_2_ (*r* = 0.45, *p* < 0.001), a significant negative correlation between FEV1 and t90 (*r* = −0.45, *p* < 0.001), and no significant relationship between FEV1 and ODI3 (*r* = −0.20, *p* = 0.076).

After dividing the study population into the two subgroups, there was a significant difference (*p* < 0.001; Student’s *t*-test) in FEV1 between Group A (89 ± 18%) and Group B (66 ± 18%).

A Mann–Whitney *U* test was performed to evaluate whether age, BMI, ODI3, avgSpO_2_, and t90 differed between the two subgroups. The results indicated that there was no significant difference between the two subgroups in terms of age (*p* = 0.071), BMI (*p* = 0.351), ODI3 (*p* = 0.883), avgSpO_2_ (*p* = 0.058), and t90 (*p* = 0.182) (see Table 2).

**Figure 3:**
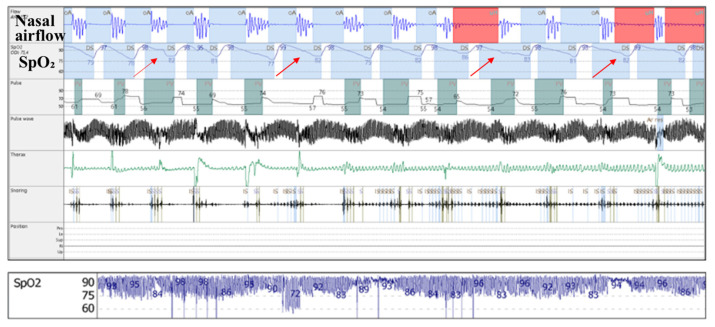
Above: 10 min epoch of cardiorespiratory polygraphy in obese patient with severe obstructive sleep apnea with saw-tooth pattern in pulse oximetry curve (red arrows): *oxyhaemoglobin saturation* (SpO_2_). Below: continuous overnight (8 h) pulse oximetry waveform of the same patient.

**Figure 4:**
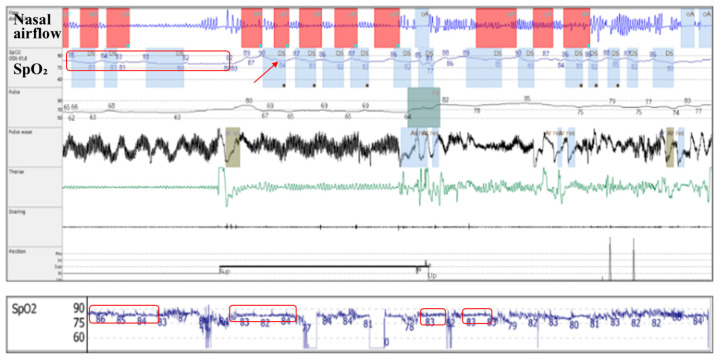
Above: 10 min epoch of cardiorespiratory polygraphy in patient with alveolar hypoventilation (plateau pattern; red box) in the context of obesity and COPD, associated with obstructive sleep apnea (saw-tooth pattern; red arrow): *oxyhaemoglobin saturation* (SpO_2_). Below: continuous overnight (7 h) pulse oximetry waveform of the same patient (plateau pattern; red box).

Concurrent with the qualitative analysis of pulse oximetry parameters, a subjective/qualitative assessment of pulse oximetry waveforms was also performed. We identified two patterns of nocturnal hypoxemia: intermittent/saw-tooth (Figure 3) and continuous/plateau (Figure 4).

## 4. Discussion

The characteristics of our patients matched those described in the literature for moderate and severe obstructive sleep apnea [20,21]: male, mean age of 60 years, and obese. A history of smoking was noted in more than half of the patients, and 14 (18%) had a positive diagnosis of COPD.

In agreement with current data [22,23], our study showed that higher BMI values were linked with a higher ODI3, a lower avgSpO_2_, and more time spent with oxyhaemoglobin saturation below 90% (t90). As expected, there was also a strong correlation between the ODI3 values and the studied parameters of nocturnal hypoxemia: a negative correlation with avgSpO_2_ and a positive correlation with t90.

Several mechanisms could explain these findings. Android fat distribution, which characterizes metabolic syndrome, with an increased hip-to-waist ratio and fat deposits around the neck, predisposes patients to upper airway narrowing and nocturnal alveolar hypoventilation [24]. Both are important mechanisms involved in the development of obstructive sleep apnea [5,25] and nocturnal hypoxemia severity and patterns, outlined as follows:

(a) Increased upper airway flow limitation, due to peripharyngeal adipose tissue accumulation, is responsible for the development of obstructive respiratory events. It is also a contributing factor to event severity (hypopnea or apnea), frequency, and duration [3]. This leads to a characteristic intermittent (saw-tooth) hypoxemia pattern (Figure 3), consisting of intermittent drops and recoveries in oxyhaemoglobin saturation. This is directly quantified using ODI3 and contributes to lower nocturnal oxyhaemoglobin saturation levels (quantified in our study using avgSpO_2_ and t90).
(b) Reduced diaphragmatic excursions, impaired chest wall compliance, and airway closure in the dependent zones of the lungs, as observed in obese patients and more severely in supine position and REM (rapid eye movement) sleep, contribute to alveolar hypoventilation, resulting in low oxyhaemoglobin saturation levels [26,27,28], with a continuous (plateau) hypoxemia pattern (Figure 4). Nocturnal hypoventilation with consecutive continuous hypoxemia and intermittent desaturations (specific to OSA) both contribute to a low avgSpO_2_ and a high t90.

Oximetric parameters have predictive value regarding overall cardiovascular mortality in obese patients with OSA. Oxyhaemoglobin saturation levels and patterns, determined by overnight pulse oximetry, may reveal the underlying mechanism involved in the nocturnal hypoxemia burden in these patients [29].

Lung function abnormalities have also been noted in obese patients in the absence of lung parenchyma impairment [1,27,30]. Adipose tissue accumulation in the bronchial wall and local adipokine production have been demonstrated in both animal [31] and human models [6]. Studies have shown a reduction in functional residual capacity and expiratory reserve volume in obese patients, requiring more work to breath to maintain adequate ventilation. In morbidly obese patients, this is further accentuated by small airway closure and air trapping, resulting in dynamic pulmonary hyperinflation [27]. Systemic inflammation secondary to the metabolic activity of adipose deposits could also be a contributing factor to lung function impairment in obese patients with normal lung parenchyma [30].

We found a significant negative relationship between BMI and FEV1 in our study population. In patients with associated COPD, we expected a more important pulmonary functional impairment, as confirmed by our analysis: patients with associated COPD had a significantly lower FEV1 mean value compared to those without COPD in the absence of a statistically significant BMI difference between the two subgroups.

After we excluded obesity (BMI) as a confounding factor, lower values of FEV1 were associated with an increased severity of nocturnal hypoxemia (lower avgSpO_2_, higher t90), probably as a result of functional impairment due to COPD. Moreover, FEV1 did not corelate with ODI3, suggesting that impaired lung function may contribute to nocturnal hypoxemia through pathophysiological mechanisms other than those seen in obstructive sleep apnea (other than intermittent/saw-tooth hypoxemia, as seen in OSA).

Considering the above, it can be concluded that COPD and alveolar hypoventilation secondary to obesity may present similar quantitative pulse oximetry patterns (a lower avgSpO_2_ and a higher t90), which would be consistent with a continuous/plateau hypoxemia pattern on the waveform analysis.

Subsequently, we investigated whether the presence of COPD could have an impact on the severity of nocturnal hypoxemia. As per our inclusion criteria, no patients had daytime hypoxemia. The average nocturnal SpO_2_ of the total study population had a mean value of 91.1%. There was a tendency towards lower average nocturnal SpO_2_ levels in patients from Group B (patients with associated COPD). Age, BMI, ODI3, and t90 did not differ significantly between the two subgroups.

Even though the test did not achieve statistical significance (*p* = 0.058), we consider this result important. The tendency towards lower nocturnal SpO_2_ levels in patients with OSA-COPD overlap (Group B), unexplained by differences in BMI or OSA severity, shows that COPD contributes to the nocturnal continuous hypoxemia pattern, even in patients with normal waking SpO_2_ levels [13]. According to our findings, nocturnal hypoxemia is more important at lower FEV1 values.

The t90 values did not differ significantly between the two subgroups. This could be attributed to the mild-to-moderate functional lung impairment observed in the studied population (patients with daytime hypoxemia were not included).

What our findings suggest is that, in patients with obstructive sleep apnea, the presence of nocturnal hypoxemia (either suggested by a low avgSpO_2_ and/or a high t90, or identified during the visual/qualitative analysis of the waveform pattern) may suggest the need to perform pulmonary function tests in order to differentiate patients with alveolar hypoventilation secondary to obesity (restrictive syndrome) from those with associated COPD (obstructive syndrome).

Assessing the different hypoxemic mechanisms present in patients with obstructive sleep apnea may have an impact on choosing the optimal therapeutic approach (positive airway pressure therapy with or without supplemental oxygen, depending on whether lung parenchymal involvement is present or not).

One limitation of our study is its modest sample size. Patients with abnormal daytime SpO_2_ levels or comorbid respiratory pathologies other than COPD, which could have acted as confounding factors in our results, were excluded from the study. Moreover, patients with mild obstructive sleep apnea (apnea–hypopnea index: 5–14 events/h) were not included.

The intermittent hypoxemia pattern, revealed by continuous overnight pulse oximetry alone, cannot distinguish between the central or obstructive respiratory events that led to this pattern, and consequently it is difficult, for now, to standardize diagnostic criteria and treatment guidelines in the absence of a nasal airflow pressure cannula [32].

## 5. Conclusions

Different phenotypes of nocturnal hypoxemia can be identified using quantitative and qualitative analyses of overnight pulse oximetry in patients referred for obstructive sleep apnea diagnosis and treatment.

The presence of waveform patterns suggestive of alveolar hypoventilation, in addition to intermittent desaturating events secondary to obstructive sleep apnea, can lead to further diagnosis tests to assess the integrity of lung parenchyma and blood gas exchange abnormalities (pulmonary function tests, arterial blood gases).

At the same time, quantitative overnight pulse oximetry parameters (i.e., hypoxic burden) are useful in stratifying patients for the risk of cardio-metabolic complications. This may have an impact on choosing the optimal therapeutic approach.

Overnight pulse oximetry could be a valuable, accessible, non-invasive tool in clinical settings for assessing the mechanisms of chronic respiratory failure, potential therapies, and their efficiency. It may also be used to document the efficiency of the proposed treatment (positive airway pressure therapy with or without supplemental oxygen).

## Figures and Tables

**Table 1:** Patient characteristics of the study population: body mass index (BMI), number of drops with ≥3% SpO_2_ per hour of recording time (ODI3), average nocturnal oxyhaemoglobin saturation (avgSpO_2_), percentage of continuous pulse oximetry evaluation time with oxyhaemoglobin saturation below 90% (t90), forced expiratory volume in the first second (FEV1), standard deviation (SD), and 25th and 75th percentile values (25th, 75th).

Study Population (n = 76)	Mean (SD)	Median (25th, 75th)
▪ Age (years)	59 (11)	62 (53, 68)
▪ BMI (kg/m^2^)	36 (6)	35 (32, 39)
▪ avgSpO_2_ (%)	91.1 (3.4)	92 (89, 93)
▪ t90 (%)	23.9 (25)	13 (3.3, 45.7)
▪ ODI3 (events/h)	46 (25)	42 (26, 58)
▪ FEV1 (% of predicted value)	85.3 (20.5)	88.2 (67.4, 102)

**Table 2:** Comparison of patient characteristics between the two subgroups: body mass index (BMI), number of drops with ≥3% SpO_2_ per hour of recording time (ODI3), average nocturnal oxyhaemoglobin saturation (avgSpO_2_), percentage of continuous pulse oximetry evaluation time with oxyhaemoglobin saturation below 90% (t90), forced expiratory volume in the first second (FEV1), standard deviation (SD), and 25th and 75th percentile values (25th, 75th).

Variable		Group A (n = 62)	Group B (n = 14)
▪ Age (years)	Mean (SD)	58 (12)	64 (7)
	Median (25th, 75th)	60 (51, 67)	67 (59, 68)
▪ BMI (kg/m^2^)	Mean (SD)	36 (6)	37 (5)
	Median (25th, 75th)	35 (31, 38)	35 (34, 40)
▪ avgSpO_2_ (%)	Mean (SD)	91.3 (3.6)	90.3 (2.5)
	Median (25th, 75th)	92.2 (90, 94)	91 (89, 92)
▪ t90 (%)	Mean (SD)	22.3 (24.7)	31 (26.1)
	Median (25th, 75th)	11.5 (3, 33.5)	24.8 (5.3, 50)
▪ ODI3 (events/h)	Mean (SD)	47 (26)	43 (20)
	Median (25th, 75th)	43 (25, 58)	40 (30, 49)
▪ FEV1 (% of predicted value)	Mean (SD)	89.5 (18.7)	66.5 (18.1)
	Median (25th, 75th)	92.1 (76.8, 104.5)	62.5 (50.2, 84)

## Data Availability

For information about the data used, please contact the corresponding author.
